# Stimulatory Role of SPAK Signaling in the Regulation of Large Conductance Ca^2+^-Activated Potassium (BK) Channel Protein Expression in Kidney

**DOI:** 10.3389/fphys.2020.00638

**Published:** 2020-07-02

**Authors:** Ye Bi, Chunmei Li, Yiqian Zhang, Yunman Wang, Shan Chen, Qiang Yue, Robert S. Hoover, Xiaonan H. Wang, Eric Delpire, Douglas C. Eaton, Jieqiu Zhuang, Hui Cai

**Affiliations:** ^1^Department of Pediatric Nephrology, The Second Affiliated Hospital/Yuying Children Hospital, Wenzhou Medical University, Wenzhou, China; ^2^Renal Division, Department of Medicine, Emory University School of Medicine, Atlanta, GA, United States; ^3^Department of Physiology, Emory University School of Medicine, Atlanta, GA, United States; ^4^Section of Nephrology, Atlanta Veterans Administration Medical Center, Decatur, GA, United States; ^5^Department of Anesthesiology, Vanderbilt University Medical School, Nashville, TN, United States

**Keywords:** BK, ERK and SPAK signaling pathway, ubiquitination, lysosomal degradation pathway, high potassium diet

## Abstract

SPS1-related proline/alanine-rich kinase (SPAK) plays important roles in regulating the function of numerous ion channels and transporters. With-no-lysine (WNK) kinase phosphorylates SPAK kinase to active the SPAK signaling pathway. Our previous studies indicated that WNK kinases regulate the activity of the large-conductance Ca^2+^-activated K^+^ (BK) channel and its protein expression via the ERK1/2 signaling pathway. It remains largely unknown whether SPAK kinase directly modulates the BK protein expression in kidney. In this study, we investigated the effect of SPAK on renal BK protein expression in both HEK293 cells and mouse kidney. In HEK293 cells, siRNA-mediated knockdown of SPAK expression significantly reduced BK protein expression and increased ERK1/2 phosphorylation, whereas overexpression of SPAK significantly enhanced BK expression and decreased ERK1/2 phosphorylation in a dose-dependent manner. Knockdown of ERK1/2 prevented SPAK siRNA-mediated inhibition of BK expression. Similarly, pretreatment of HEK293 cells with either the lysosomal inhibitor bafilomycin A1 or the proteasomal inhibitor MG132 reversed the inhibitory effects of SPAK knockdown on BK expression. We also found that there is no BK channel activity in PCs of CCD in SPAK KO mice using the isolated split-open tubule single-cell patching. In addition, we found that BK protein abundance in the kidney of SPAK knockout mice was significantly decreased and ERK1/2 phosphorylation was significantly enhanced. A high-potassium diet significantly increased BK protein abundance and SPAK phosphorylation levels, while reducing ERK1/2 phosphorylation levels. These findings suggest that SPAK enhances BK protein expression by reducing ERK1/2 signaling-mediated lysosomal and proteasomal degradations of the BK channel.

## Introduction

Pseudohypoaldosteronism type II (PHAII), characterized by hypertension, hyperkalemia, and metabolic acidosis (Hadchouel et al., 2016), results from mutations in WNK kinase family members WNK1 and WNK4 (Wilson et al., 2001). PHAII is also caused by mutations in *Cullin 3* and *Kelch-like 3*, components of a ubiquitin ligase that degrades WNK kinases. The renal distal nephron contains two major potassium channels, ROMK (also known as Kir1.1 or KCNJ1) and BK (big potassium, Maxi-K, or Slo) channels (Bailey et al., 2006). The BK channels, which are expressed in the apical membrane of intercalated cells and principal cells (Welling, 2016), are large-conductance, voltage- and calcium-activated potassium channels responsible for “flow-induced potassium secretion” (Woda et al., 2001; Grimm and Sansom, 2007). BK channels consist of alpha, beta, and gamma subunits. The alpha subunit functions as an independent channel. Several studies have shown that members of the WNK family are involved in the regulation of BK channels (Zhuang et al., 2011; Liu et al., 2015).

SPAK (SPS1-related proline/alanine-rich kinase) is serine/threonine kinase regulated by WNK kinase (Yang et al., 2010; Gagnon and Delpire, 2012). It is phosphorylated by WNK kinase and thus facilitates regulation of ion transport and blood pressure (O’Reilly et al., 2003; Glover et al., 2011). Previous studies have shown that SPAK kinase is expressed in renal epithelial cells including proximal tubule, limb of the loop of Henle, distal convoluted tubule (DCT), connecting tubule, and collecting ducts (including principal cells and intercalated cells) in the kidney (Ferdaus et al., 2017; Wu et al., 2019). SPAK not only participates in the regulation of ion channels (Falin et al., 2011; Warsi et al., 2014c; Ahmed et al., 2015) but also regulates a variety of carriers, including NaCl (NCC) and Na^+^-K^+^-2Cl^–^ (NKCC) cotransporters, KCl symporters, and Na^+^/H^+^ exchangers (Delpire and Gagnon, 2006; Gagnon et al., 2006; Vitari et al., 2006; Geng et al., 2009; Gagnon and Delpire, 2010). In previous studies, we showed that multiple members of the WNK family regulate BK protein expression via the ERK1/2 pathway (Zhuang et al., 2011; Liu et al., 2015) or explored the effects of SPAK and ERK1/2 in the NCC regulation process in the WNK family (Zhou et al., 2012; Feng et al., 2015; Wang et al., 2016). Although SPAK is widely expressed in the renal tubules, the link between SPAK and BK remains largely unknown. Therefore, we hypothesized that SPAK, also controlled by the WNK family, is like ERK1/2 and has a function of regulating BK protein expression.

Here, we report data showing that SPAK influences BK by altering degradation pathways and enhancing ERK activity. These data indicate that SPAK can increase the expression of BK protein by inhibiting the ERK1/2 signal pathway and reducing the degradation of BK by both lysosomal and proteasomal pathways.

## Materials and Methods

### Experimental Animals and Dietary Manipulation

The Institutional Animal Care and Use Committees from Emory University and the Atlanta Veterans Affairs Medical Center approved all animal-related procedures. All male mice were 8–10 weeks old weighing 25–30 g. The generation of SPAK KO (SPAK −/−) mice has been described in detail previously (Geng et al., 2009). The cortical parts of the kidneys from C57BL/6 mice and SPAK KO mice were used for Western blot analysis. The C57BL/6 mice were assigned to different dietary groups: (Hadchouel et al., 2016) control K group: four mice were fed with control potassium diet (control K; 1% KCl, TD #88238) and (Wilson et al., 2001) HK group: four mice were fed with high-potassium diet (HK; 10% KCl, TD #09075). All diets were purchased from Harlan Laboratories. The animals were fed with these diets for 10 days and then euthanized by cervical dislocation after isoflurane anesthesia, and kidneys removed and studied. Part of the kidney cortex was used for Western blot analysis and the remaining kidney cortex was stored at −80°C.

### Plasmids and Constructs

Human SPAK was amplified by PCR using a human kidney cDNA library as the template. Myc-tagged wild-type (WT) rabbit Maxi K plasmid (BK alpha subunit) was a gift from Dr. William Guggino (Johns Hopkins University, Baltimore, MD, United States). The WT SPAK plasmid contains a flag-tag. All constructs were confirmed by DNA sequencing. These constructs have been successfully expressed in the HEK293 cells, as confirmed by Western blot analysis.

### Cell Culture and Transfection

HEK293 cells were maintained in DMEM medium containing 10% FBS, 1% penicillin (100 U/ml), and 1% streptomycin (100 U/ml). Lipofectamine 2000 (Invitrogen, 11668019) was used for transfection according to the manufacturer’s instructions. Cells were transfected with a series of doses of flag-SPAK plasmid and the same dose of myc-BK plasmid (0.6 μg), and 48 h later, the cell lysate was used for Western blot analysis. The expression of SPAK or ERK1/2 was knocked down in HEK293 cells using siRNA. SPAK siRNA (Santa Cruz Biotechnology, [h], Sc-76547), ERK1/2 siRNA (Cell Signaling Technology, 6560s), or control siRNA-A (Santa Cruz Biotechnology, sc-37007) was transfected into HEK-293 cells with Lipofectamine RNAiMax (Invitrogen, 13778150). The cells were transfected with siRNA overnight, the same dose of myc-BK plasmid was transfected the next day, and the cells were lysed after 48 h and subjected to Western blot analysis.

### Western Blot Analysis

Cells were harvested and processed as previously described (Cai et al., 2006; Zhuang et al., 2011; Wang et al., 2016). Briefly, protease inhibitors (1 tablet per 50 ml of solution, Roche Diagnostics GmbH, 11873580001) and sodium orthovanadate 80 μM (Sigma, s6508) are added to a RIPA lysis buffer for lysing cells transiently transfected with various DNA constructs or siRNAs. After fully lysing for 30 min, the lysates were centrifuged at 13200 rpm for 30 min to precipitate insoluble material. The mouse kidney cortex was lysed in Tissue Protein Extraction Reagent (T-PER, Thermo, 78510) or RIPA lysis buffer supplemented with Halt protease and phosphatase inhibitor cocktail (Thermo, 78442). The kidney lysates were centrifuged at 6000 rpm or 12000 rpm for 30 min to precipitate insoluble material. The proteins from the supernatant were quantified using a Pierce BCA Protein Assay kit (Pierce, Rockford, IL, United States). After mixing with Laemmli buffer (Bio-Rad) and incubating at 100°C for 3 min or 42°C for 15 min, the protein samples were separated by SDS-PAGE and electrophoretically transferred to polyvinylidene difluoride (PVDF) membranes (Bio-Rad) for Western blot. The probing with specific antibodies and subsequent detection with Clarity Western ECL substrate (Bio-Rad) or Super signal (Pierce) were performed according to standard procedures as described previously (Gagnon et al., 2006; Wang et al., 2016). The antibodies used were anti-actin (EMD Millipore, MBA1501), anti-phospho-p44/42 MAPK (Erk1/2) (Thr202/Tyr204), and anti-p44/42 MAPK (ERK 1/2) (Cell Signaling Technology, 9101L/9102L), anti-phospho-SPAK (Ser373)/phospho-OSR1 (Ser325) (EMD Millipore, 07-2273), anti-t-SPAK (Santa Cruz Biotechnology, sc-517361), anti-myc-Tag (Cell Signaling Technology, #2276s), and anti-BKα (Alomone Labs, APC-021).

### Single-Channel Recording in Isolated, Split-Opened Kidney Tubules

Renal tubules were manually dissected in cold Hanks’ balance salt solution, and the CCD was identified by morphology. Tubules were placed in extracellular bathing solution (150 NaCl, 5 KCl, 10 CaCl_2_, 2 MgCl_2_, 5 glucose, and 10 HEPES, pH adjusted to 7.4) in a plastic dish before being split open to reveal the apical surface of the cells before single-channel patch-clamp as previously described (Liu et al., 2015; Zou et al., 2018). Briefly, a microelectrode (pipette) was filled with physiological buffer solution (150 KCl, 2 MgCl_2_, and 10 HEPES, pH 7.3) and lowered to a single cell before application of a small amount of suction to achieve a >1-G seal. The channel currents were filtered at 1 kHz, recorded with an AxoPatch 1D amplifier (Molecular Devices), and sampled at 5 kHz. Channel activity (NPo) was calculated from pClampfit 9.2 data analysis software (Molecular Devices). Channel number (N) was determined from the maximal number of transitions during 10–20 min of recording, and channel Po was calculated as the ratio of NPo to N.

### Statistical Analysis

The data are presented as mean ± SD. Western blot band densities were scanned and quantified using ImageJ software. Statistical significance was determined by *t*-test when two groups were compared or either one-way ANOVA or two-way ANOVA, followed by Bonferroni’s *post hoc* tests when multiple groups were compared. We assigned significance at *p* < 0.05.

## Results

### Effect of SPAK on BK Protein Expression

Our previous studies showed that WNK kinase affects the expression of BK protein through the ERK1/2 signaling pathway (Zhuang et al., 2011; Liu et al., 2015). WNK kinase is also known to phosphorylate SPAK kinase to activate the SPAK signaling pathway. In SPAK KO mice, ERK 1/2 phosphorylation is enhanced (Feng et al., 2015). We therefore hypothesized that SPAK kinase might affect BK protein expression by modulating ERK 1/2 phosphorylation.

**FIGURE 1:**
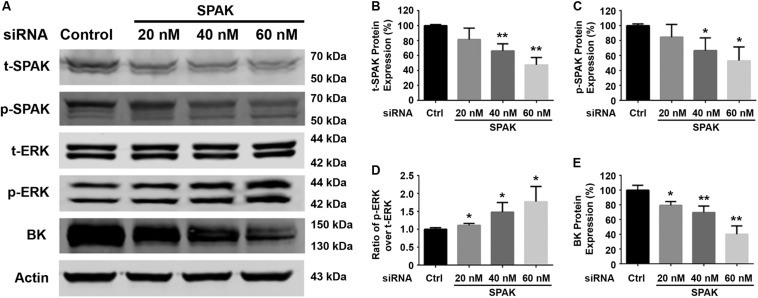
Knockdown of SPAK expression reduced BK protein expression and increased ERK 1/2 phosphorylation in HEK293 cells. HEK 293 cells were transfected with control siRNA or a series of doses of siRNA SPAK overnight, and the cells were transfected with myc-BK plasmids the next day as indicated. Forty-eight hours after transfection, cells were lysed and subjected to SDS-PAGE and Western blot analysis. **(A)** Representative immunoblots are shown for BK, phosphorylated and total SPAK protein, phosphorylated and total ERK1/2 protein, and actin levels. **(B–E)** Bar graphs represent the average band densities of t-SPAK, p-SPAK, ratio of p-ERK1/2 over t-ERK1/2, and BK. *n* = 4; *p < 0.05, **p < 0.01 compared with the control.

**FIGURE 2:**
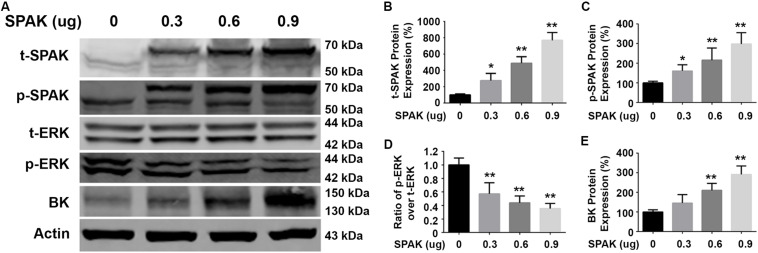
Overexpression of SPAK increased BK protein expressions while reducing ERK 1/2 phosphorylation in HEK293 cells. HEK293 cells were transfected with both pCMV-myc-BK and a series of doses of flag-SPAK plasmids indicated. Forty-eight hours after transfection, cells were lysed and subjected to SDS-PAGE and Western blot analysis. **(A)** Representative immunoblots are shown for BK, phosphorylated and total SPAK protein, phosphorylated and total ERK1/2 protein, and actin levels. **(B–E)** Bar graphs represent the average band densities of t-SPAK, p-SPAK, ratio of p-ERK1/2 over t-ERK1/2, and BK. *n* = 4; *p < 0.05, **p < 0.01 compared with the control group.

We first determined the effect of siRNA SPAK on BK protein expression in HEK293 cells transfected with myc-BK. As shown in Figure 1, SPAK expression was significantly reduced by siRNA SPAK as expected. Knockdown of SPAK expression also significantly reduced BK protein expression in a dose-dependent manner (100% ± 3.4% with siRNA SPAK at 0 nM, 79.4% ± 3.0% at 20 nM, 69.6% ± 5.1% at 40 nM, 40.1% ± 6.5% at 60 nM; *n* = 4; *p* < 0.05 compared with SPAK at 0 nM group). We then performed the SPAK overexpression experiments. As shown in Figure 2, in HEK293 cells transfected with myc-BK, SPAK overexpression significantly increased BK protein expression in a dose-dependent manner. These data indicated that SPAK significantly increases BK protein expression (100% ± 6.4% with SPAK at 0 μg, 145.5% ± 25.0% at 0.3 μg, 210.4% ± 20.6% at 0.6 μg, and 291.4% ± 25.1% at 0.9 μg; *n* = 4; *p* < 0.05 compared with SPAK 0 μg group). In addition, in HEK293 cells transfected with SPAK, SPAK overexpression significantly reduced ERK1/2 phosphorylation in a dose-dependent manner (p-ERK1/2/t-ERK1/2 ratio of 1.0 ± 0.1 with SPAK at 0 μg, 0.57 ± 0.1 at 0.3 μg, 0.44 ± 0.1 at 0.6 μg, 0.35 ± 0.04 at 0.9 μg; *n* = 4; ^∗^*p* < 0.05 compared with 0 μg SPAK group) (Figure 2), whereas knockdown of SPAK expression significantly increased ERK1/2 phosphorylation in a dose-dependent manner, as shown in Figure 1 (p-ERK1/2/t-ERK1/2 ratio of 1.0 ± 0.02 with siRNA control, 1.11 ± 0.03 with siRNA SPAK 20 nM, 1.47 ± 0.14 at 40 nM, 1.77 ± 0.21 at 60 nM; *n* = 4; ^∗^*p* < 0.05 compared with siRNA control group). The data suggested that SPAK signaling stimulates BK protein expression by inhibiting the ERK1/2 signal pathway.

### Effect of ERK 1/2 Signaling Pathway on SPAK-Mediated Regulation of BK

**FIGURE 3:**
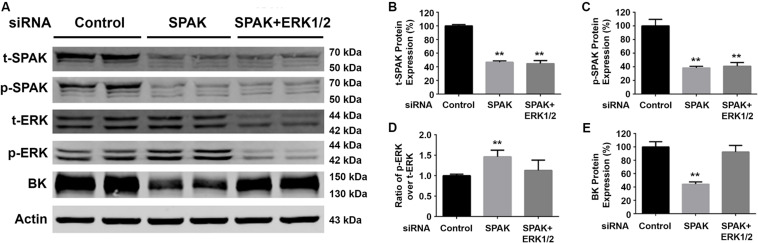
Knockdown of ERK1/2 expression abolished the SPAK siRNA-mediated inhibition of BK protein expression. HEK293 cells were transfected with either siRNA control or siRNA SPAK (50 nM) with or without siRNA ERK1/2 (30 nM) as indicated. Forty-eight hours after transfection, cells were lysed and subjected to SDS-PAGE and Western blot analysis. **(A)** Representative immunoblots are shown for BK, phosphorylated and total SPAK protein, phosphorylated and total ERK1/2 protein, and actin levels. **(B–E)** Bar graphs represent the average band densities of t-SPAK, p-SPAK, ratio of p-ERK1/2 over t-ERK1/2, and BK. *n* = 4; *p < 0.05, **p < 0.01 compared with the control group.

To confirm that SPAK signaling stimulates BK protein expression through modulating the ERK1/2 signaling pathway, we performed ERK 1/2 knockdown experiments simultaneously. Western blotting was performed for t-SPAK (Figure 3A), which was quantified in Figure 3B. In HEK293 cells co-transfected with siRNA SPAK (50 nM) with or without siRNA ERK 1/2 (30 nM), SPAK expression was significantly reduced by >50% (Figures 3B,C). BK protein expression was significantly reduced (44.2% ± 1.9%) in the siRNA SPAK-transfected group compared with the siRNA control group (100% ± 4.5%; *n* = 4; ^∗∗^*p* < 0.01), while increasing ERK 1/2 phosphorylation. When in HEK293 cells co-transfected with both siRNA SPAK (50 nM) and siRNA ERK 1/2 (30 nM), ERK 1/2 protein expression and ERK1/2 phosphorylation were remarkably reduced (p-ERK1/2/t-ERK1/2 ratio of 1.46 ± 0.1 versus 1.0 ± 0.02; *n* = 4; ^∗∗^*p* < 0.01), as expected (Figure 3D). BK protein expression did not differ from controls with simultaneous SPAK and ERK 1/2 siRNA knockdown (92.4% ± 5.5%), suggesting that knockdown of ERK 1/2 abolished the effect of SPAK on BK. These data further supported that SPAK modulates BK through an ERK1/2 signaling-dependent pathway.

### Effect of SPAK Signaling on BK Protein Expression Through Both Lysosomal and Proteasomal Degradation Pathways

**FIGURE 4:**
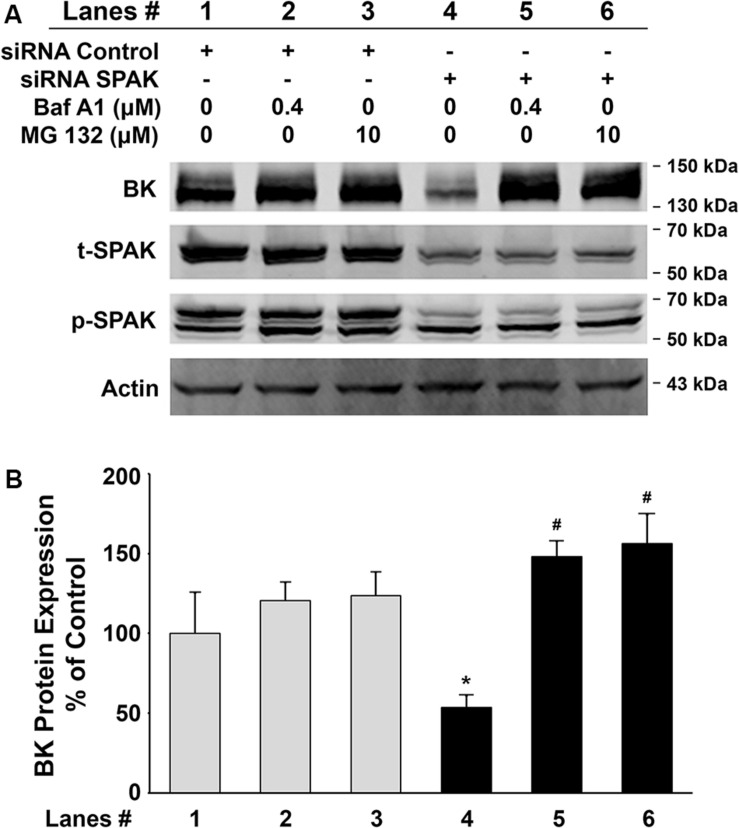
Effects of the lysosomal inhibitor, bafilomycin A1 (Baf A1), and proteasomal inhibitor, MG 132, on BK protein expressions in HEK 293 cells. HEK293 cells were transfected with BKα plasmid in combination with either control siRNA or siRNA SPAK. Sixteen hours before cell lysis, a dose of either Baf A1 (0.4 μM) or MG 132 (10 μM) was added to the culture medium. Forty-eight hours after transfection, cells were lysed and subjected to SDS-PAGE and Western blot analysis. **(A)** Representative immunoblots for BK, phosphorylated and total SPAK protein, and actin levels. Lanes 1–3 indicate the control siRNA group treated with Baf A1 (0.4 μM) and MG 132 (10 μM). Lanes 4–6 indicate the siRNA SPAK group treated with Baf A1 (0.4 μM) and MG 132 (10 μM). **(B)** Bar graph summarized from four independent experiments. Knockdown of SPAK expression (lane 4) significantly reduced total BK protein expression compared with the control group (lane 1). *n* = 4; ^#^*p* < 0.05. Both Baf A1 and MG132 treatments reversed the inhibitory effects by knocking down SPAK expression (lanes 5 and 6). *n* = 4; *p < 0.05 compared with the untreated siRNA SPAK group (lane 4). These results indicate that the inhibitory effect of SPAK siRNA on BK protein expression is abolished by both Baf A1 and MG132.

To further investigate whether inhibition of BK protein expression by SPAK knockdown is due to increased degradation, we assessed the BK protein expression levels in HEK 293 cells transfected with BKα plasmid in combination with either control siRNA or siRNA SPAK then treated with bafilomycin A1 (Baf A1) 16 h before cell lysis. Baf A1 is a lysosomal inhibitor that affects the acidic protease by disturbing the pH of the lysosome and ultimately disrupts the lysosomal degradation pathway. In Figure 4, BK protein expression was significantly reduced in HEK293 cells transfected with siRNA SPAK (50 nM) (53.5% ± 7.4% versus 100% ± 25.5% in siRNA control; *n* = 4; ^#^*p* < 0.05). However, in the group transfected with siRNA SPAK, the expression of BK protein was significantly increased after the pretreatment of HEK293 cells with Baf A1 (147.9% ± 9.8% at Baf A1 0.4 μM versus 53.5% ± 7.4% at Baf A1 0 μM; *n* = 4; ^∗^*p* < 0.01). In addition, we performed a similar experiment pretreatment with proteasomal inhibitor, MG 132 (10 μM). In the group transfected with siRNA SPAK, the expression of BK protein was significantly increased after the pre-incubation of HEK293 cells with MG 132 (156.2% ± 18.1% at MG132 10 μM versus 53.5% ± 7.4% at MG132 0 μM; *n* = 4; ^∗^*p* < 0.05). These data indicated that stimulation of SPAK signaling increases BK protein expression by stabilizing BK protein and preventing BK from degradation through both lysosomal and proteasomal pathways.

### Effects of SPAK Knockout on BK Protein Abundance and ERK1/2 Phosphorylation in Both WT and SPAK KO Mice

**FIGURE 5:**
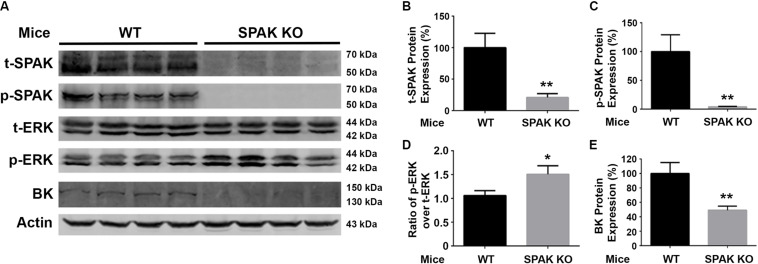
BK protein abundance is significantly reduced while increasing ERK1/2 phosphorylation in SPAK KO mice. **(A)** Representative immunoblots are shown for BK, phosphorylated and total SPAK protein, phosphorylated and total ERK1/2 protein, and actin levels in both WT and SPAK KO mice. **(B–E)** Bar graphs represent the average band densities of t-SPAK, p-SPAK, ratio of p-ERK1/2 over t-ERK1/2, and BK. *n* = 4; *p < 0.05, **p < 0.01 compared with the WT mouse group.

We further investigated the effects of SPAK knockout on the phosphorylation of ERK1/2 and the expression of BK protein abundance in both WT and SPAK KO mice. As shown in Figure 5, the level of ERK1/2 phosphorylation was significantly increased in SPAK KO mice compared to WT mice (p-ERK1/2/t-ERK1/2 ratio of 1.5 ± 0.07 versus 1.0 ± 0.04; *n* = 4; ^∗∗^*p* < 0.01), while the level of BK protein abundance was significantly reduced (49.2% ± 2.8% versus 100% ± 7.6% in WT mice; *n* = 4; ^∗∗^*p* < 0.01). These data are consistent with the findings obtained from experiments in cells and further confirmed that SPAK signaling stimulates the BK protein expression by inhibiting ERK1/2 phosphorylation.

### Effect of SPAK on BK Channel Activity in SPAK KO Mice

**FIGURE 6:**
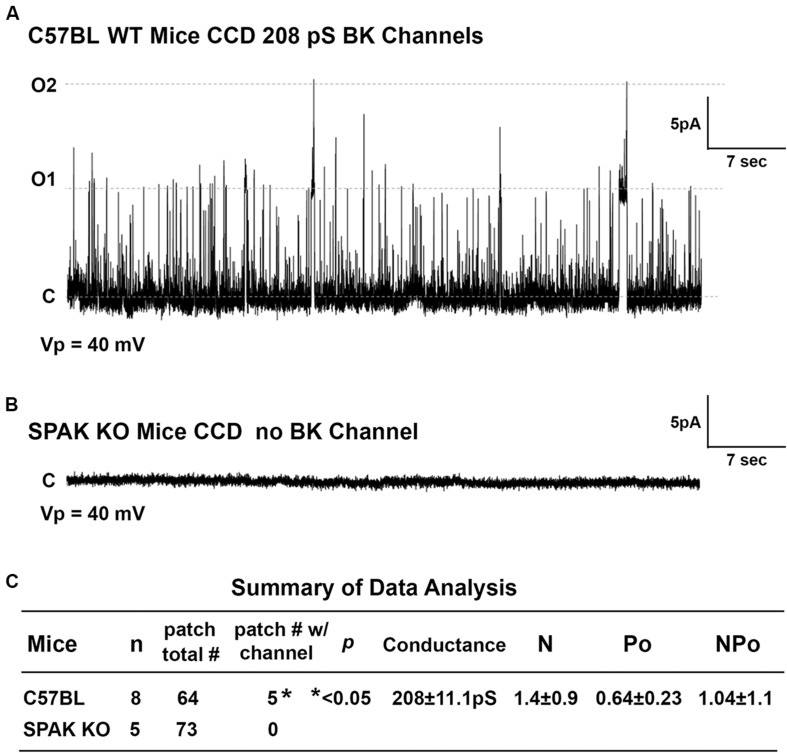
The effect of SPAK gene deletion on BK channel activity in the principal cells (PCs) of cortical collecting ducts (CCD) isolated from WT and SPAK KO mice. WT and SPAK KO mice were sacrificed, and renal tubules were manually dissected. BK channel activity was recorded from cell-attached single-cell patches on PCs of CCD. **(A,B)** The representative recording traces in PCs of CCD from WT and SPAK KO mice groups at a holding potential (Vp) of 40 mV, respectively. **(C)** Summary of data analysis. As shown in **(C)**, in WT mice (*n* = 8), the fraction of BK channel # over total patches is 0.78 (5 out of 64), whereas in SPAK KO mice (*n* = 5), the fraction of BK channel # over total patches is less than 0.001 (0 out 73), *p* < 0.05. These data suggested that BK channel activity is markedly reduced and absent in SPAK KO mice compared to WT mice. Note: N: channel number; Po: channel open probability; and NPo: channel activity.

To determine whether SPAK gene deletion affects BK channel activity in SPAK KO mice, we have performed the isolated split-open tubule single-cell patching in the principal cells (PCs) of cortical collecting duct (CCD) isolated from SPAK KO and WT mice, in which SPAK is normally expressed (Ferdaus et al., 2017; Wu et al., 2019). In WT mice, we found that the fraction of BK channels/# of patches for WT is 0.78, whereas in SPAK KO mice, the fraction of BK channels/# of patches for WT is less than 0.001 (*p* < 0.5), as shown in Figure 6. These data suggested that BK channel activity is markedly reduced and almost absent in SPAK KO mice. Interestingly, we did see ROMK channel activity is increased in SPAK KO mice (data not shown), suggesting a likely compensated effect, also in the absence of the BK channel. These data suggest that SPAK signaling is involved in regulating BK channel activity in the PCs.

### Effect of High Dietary Potassium on the Expressions of SPAK, BK Protein, and ERK 1/2 Phosphorylation in Mice

**FIGURE 7:**
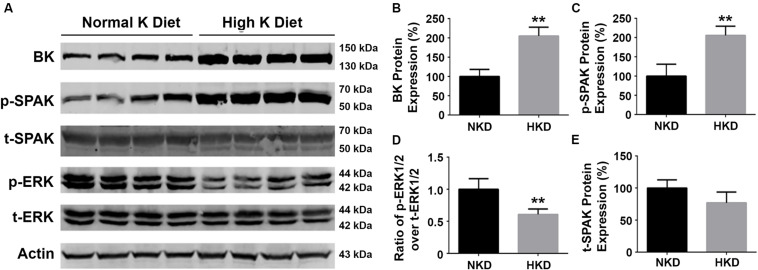
The effects of high-potassium diet on protein levels of BK, SPAK phosphorylation, and ERK1/2 phosphorylation in WT mice. WT mice were fed with either normal K (NK, 1%) diets or high K (HK, 10%) diets for 10 days. The kidneys were then harvested for Western blot analysis. **(A)** Representative immunoblots are shown for BK, phosphorylated and total SPAK protein, phosphorylated and total ERK1/2 protein, and actin levels in both NK and HK mice groups. **(B–E)** Bar graphs represent the average band densities of BK, p-SPAK, t-SPAK, and the ratio of p-ERK1/2 over t-ERK1/2. Compared with the NK group, the BK protein abundance in the HK group was significantly increased. The phosphorylation level of SPAK was significantly increased in the HK group, and the phosphorylation level of ERK1/2 was significantly reduced compared to the NK group. *n* = 4; *p < 0.05, **p < 0.01.

Our previous studies have shown that a HK diet increased BK channel expression in the kidney and that this was associated with a decrease in ERK1/2 phosphorylation status (Liu et al., 2015). To determine whether SPAK signaling is involved in the regulation of BK protein, we investigated the effects of an HK (10%) diet (10 days) on the levels of BK protein, and phosphorylation levels of SPAK and ERK signaling in WT mice. As shown in Figure 7, the levels of BK protein abundance (205.1% ± 11.4% versus 100% ± 9.2%; *n* = 4; ^∗∗^*p* < 0.01) and the levels of SPAK phosphorylation (205.8% ± 11.9% versus 100% ± 15.4%; *n* = 4; ^∗∗^*p* < 0.01) in the HK group were significantly increased compared with the normal potassium (NK) diet group, whereas the levels of ERK 1/2 were significantly decreased in the HK diet group compared to the NK diet group (p-ERK1/2/t-ERK1/2 ratio of 0.6 ± 0.04 versus 1.0 ± 0.08; *n* = 4; ^∗∗^*p* < 0.01). These data indicated that HK diet increases the BK protein abundance by increasing SPAK phosphorylation and inhibiting ERK1/2 phosphorylation, which further support the note that SPAK signaling plays a stimulatory role in BK regulation by interacting with and inhibiting the ERK 1/2 signaling pathway.

## Discussion

In the present study, we report that overexpression of SPAK in HEK293 cells transiently transfected with myc-BK significantly reduced ERK1/2 phosphorylation while significantly enhancing BK protein expression. The knockdown of SPAK expression not only increased ERK1/2 phosphorylation but also significantly inhibited BK protein expression. The knockdown of ERK1/2 disrupted the inhibition of BK expression by siRNA SPAK. Both lysosomal inhibitor bafilomycin A1 and proteasomal inhibitor MG 132 also reversed the inhibitory effect of siRNA SPAK on BK protein expression. *In vivo* mouse studies indicate that ERK1/2 phosphorylation and BK protein expression in SPAK KO mice are consistent with cultured cell experiments. We also found that there is no BK channel activity in PCs of CCD in SPAK KO mice using the isolated split-open tubule single-cell patching. Protein abundance of BK and SPAK phosphorylation were significantly higher, and ERK1/2 phosphorylation levels were reduced in WT mice fed a high-potassium diet. We have demonstrated that SPAK signaling has stimulatory effects on BK protein expression likely via the SPAK-mediated ERK 1/2 signaling pathway.

SPAK and oxidative stress response kinase (OSR1), two serine/threonine (S/T) kinases belonging to the germinal center-like kinase subfamily VI (Ushiro et al., 1998; Dan et al., 2001; Chen et al., 2004), are involved in various physiological processes including cell differentiation, transformation, proliferation, cytoskeletal rearrangement, and regulation of ion transport and blood pressure (Delpire and Gagnon, 2006; Gagnon and Delpire, 2012). SPAK and OSR1 are ubiquitously expressed in many organs, such as heart, brain, liver, kidney, and lung (Johnston et al., 2000; Piechotta et al., 2003). Previous studies reported that SPAK is abundantly expressed in tissues such as the cerebral cortex and kidney including renal epithelial tubules along the nephron (Ferdaus et al., 2017; Wu et al., 2019), whereas several studies show significant expression and roles for OSR1 in TAL and DCT. OSR1 is also expressed more than SPAK in cardiac tissues. It is generally accepted that SPAK can be phosphorylated by WNK (O’Reilly et al., 2003; Vitari et al., 2005, 2006; Delpire and Gagnon, 2006; Rafiqi et al., 2010; Glover et al., 2011) and then regulate a variety of ion transporters (Fezai et al., 2014, 2015b; Warsi et al., 2014a, b) and channels (Falin et al., 2011; Elvira et al., 2014, 2016; Warsi et al., 2014c; Ahmed et al., 2015; Fezai et al., 2015a), such as sodium chloride cotransporter (NCC) and sodium potassium chloride cotransporter (NKCC), potassium chloride cotransporter (KCl), and Na^+^/H^+^ exchanger (NHE). BK acts as a K^+^ channel that plays a major regulatory role in K secretion in the kidney, which is responsible for flow-dependent K^+^ secretion, and it is also involved in the regulation of blood pressure. In the present study, we found for the first time that SPAK has a stimulatory role in BK regulation. Knockdown of SPAK expression decreases BK protein expression, whereas SPAK overexpression enhances BK protein expression. The tubular split-open single-cell patching recording showed that BK channel activity in PCs of CCD isolated from SPAK KO mice is almost absent, suggesting that SPAK signaling has a stimulatory effect on BK channel activity. Interestingly, we did see that likely ROMK channel activity is increased in SPAK KO mice in the PCs, suggesting a likely compensated effect, also in the absence of the BK channel, which could explain why SPAK KO mice did not exhibit a hyperkalemia phenotype, rather than hypokalemia, one of the Gitelman syndrome phenotypes. A previous study using DCT-1 SPAK constitutive-active (CA-SPAK) knock-in mice showed that BK alpha subunit protein abundance from the renal cortex is increased, whereas ROMK protein abundance is significantly decreased (Grimm et al., 2017). In the present study, BK protein expression is downregulated in SPAK knockdown cells and in SPAK KO mice and BK channel activity is almost absent in PCs of CCD isolated SPAK KO mice, indicating a stimulatory effect of SPAK on BK. However, the exact mechanism underlying the SPAK-mediated regulation of BK channel activity and protein expression in the renal tubules remains to be further explored. Further study is needed to investigate whether SPAK signaling modulates BK channel activity directly vs. indirectly through intermediate players or the compensatory use of generation of PC-specific SPAK KO mice to test the role of SPAK kinase in BK regulation in PCs of CCD in the future.

However, our findings are in contrast to the previous report in which SPAK was shown to inhibit BK activity in *Xenopus* oocytes. In this report, investigators used the Ca^2+^-insensitive BK channel mutant (BK^M513*I+*Δ^ ^899–903^) for electrophysiological experiments in which cRNA for the BK mutant was injected into oocytes and was used to perform patch clamp experiments. Since BK is a calcium- and voltage-activated K channel, the Ca-insensitive BK mutant might exhibit a different channel activity in response to SPAK signaling. In the present study, WT BK constructs have been used for all the experiments, which might explain why findings from the present study are different from the previous report (Elvira et al., 2016). Whether the calcium sensor domain in the BK channel confers different responses of BK to SPAK kinase remains to be explored.

The extracellular signal-regulated kinase 1/2 (ERK1/2) mitogen-activated protein kinase (MAPK) signaling pathway regulates many cellular functions, usually phosphorylated and activated as part of the Ras/Raf/MEK1/2/ERK1/2 signaling cascade following stimulation by a variety of factors (Nishimoto and Nishida, 2006; Raman et al., 2007). Previous studies have demonstrated that WNK4 inhibits BK channel activity by activating the ERK 1/2 signaling pathway and leading to enhancing BK degradation through a lysosomal pathway (Kahle et al., 2003; Zhuang et al., 2011; Wang et al., 2013; Yue et al., 2013), whereas WNK1 stimulates BK activity by suppressing the ERK 1/2 signaling pathway (Liu et al., 2015). A high-potassium (K) diet load increases BK channel activity and maximizes kaliuresis for maintaining potassium homeostasis. BK protein abundance is also increased after high dietary K load in mice (Cornelius et al., 2015; Liu et al., 2015). The present study also found that pretreatments with both lysosomal inhibitor bafilomycin A1 and proteasomal inhibitor MG 132 reverse the inhibitory effect of siRNA SPAK on BK expression, suggesting that SPAK activates BK channel activity by reducing its degradation via both lysosomal and proteasomal pathways. Since bafilomycin A1 also affects autophagy, whether the degradative pathway underlying the effect of SPAK on BK involving the autophagy process remains to be further explored. In a previous study, we found that G protein pathway suppressor 2 (GPS2) knockdown enhances BK ubiquitination and subsequently increases BK degradation, suggesting that SPAK could affect BK ubiquitination and lead to its degradation (Zhuang et al., 2018). A previous study reported that total SPAK and phosphorylated SPAK are significantly increased in WT mice fed with a low-potassium diet (15–30 ppm K^+^) for 4 days (Wade et al., 2015). In the present study, there was a trend for total SPAK to decrease in mice fed with 10 days of HK (10% KCl) diet but does not reach statistical difference. However, phosphorylated SPAK is significantly increased in mice fed with 10 days of HK diet. These data suggested that dietary K intakes modulate the SPAK signaling pathway. At the same time, BK protein abundance is significantly enhanced in the HK-fed mice, which is closely associated with a decrease in ERK1/2 phosphorylation and enhanced SPAK phosphorylation in the HK-fed mice, suggesting that HK diets increase BK protein abundance through modulating both ERK1/2 and SPAK signaling pathways. We also found that in SPAK KO mice, levels of ERK1/2 phosphorylation are significantly increased, and BK expression is significantly decreased. These data show the consistence of BK levels between cellular expressions and SPAK KO mice, further supporting that SPAK promotes BK function by inhibiting the ERK 1/2 signaling pathway. In our study, SPAK signaling has been shown to modulate ERK 1/2 signaling, ultimately leading to an alteration in BK expression. When we simultaneously knocked down the ERK 1/2 signaling pathway, SPAK did not have effects on BK anymore (Figure 3), suggesting that ERK 1/2 lies downstream of SPAK in BK regulation. However, it is still unclear how modulating SPAK signaling affects ERK 1/2 phosphorylation, which needs to be further explored in the future study.

## Conclusion

Our data demonstrated for the first time that SPAK upregulates BK protein expression and is mediated by the ERK1/2 signaling pathway to reduce BK degradation through both lysosomal and proteasomal pathways. These data suggest that SPAK kinase plays an important role in regulating BK, particularly in principal cells of CCD, which helps maintain potassium homeostasis.

## Data Availability Statement

The datasets generated for this study are available on request to the corresponding author.

## Ethics Statement

The animal study was reviewed and approved by The Institutional Animal Care and Use Committees from Emory University and the Atlanta Veterans Affairs Medical Center approved all animal-related procedures.

## Author Contributions

JZ and HC designed the study and wrote the manuscript. YB, CL, YZ, YW, SC, and QY performed the experiments. YB, CL, and DE analyzed the data. RH, XW, ED, and DE gave technical support, conceptual advice and reviewed the manuscript. All authors contributed to the article and approved the submitted version.

## Conflict of Interest

The authors declare that the research was conducted in the absence of any commercial or financial relationships that could be construed as a potential conflict of interest.
